# Biased Influences of Low Tumor Purity on Mutation Detection in Cancer

**DOI:** 10.3389/fmolb.2020.533196

**Published:** 2020-12-23

**Authors:** Jun Cheng, Jun He, Shanshan Wang, Zhangxiang Zhao, Haidan Yan, Qingzhou Guan, Jing Li, Zheng Guo, Lu Ao

**Affiliations:** ^1^Department of Bioinformatics, Fujian Key Laboratory of Medical Bioinformatics, Key Laboratory of Ministry of Education for Gastrointestinal Cancer, The School of Basic Medical Sciences, Fujian Medical University, Fuzhou, China; ^2^Department of Systems Biology, College of Bioinformatics Science and Technology, Harbin Medical University, Harbin, China

**Keywords:** tumor purity, gastric cancer, microsatellite instability status, mutation calling algorithms, number of mutation

## Abstract

The non-cancerous components in tumor tissues, e.g., infiltrating stromal cells and immune cells, dilute tumor purity and might confound genomic mutation profile analyses and the identification of pathological biomarkers. It is necessary to systematically evaluate the influence of tumor purity. Here, using public gastric cancer samples from The Cancer Genome Atlas (TCGA), we firstly showed that numbers of mutation, separately called by four algorithms, were significant positively correlated with tumor purities (all *p* < 0.05, Spearman rank correlation). Similar results were also observed in other nine cancers from TCGA. Notably, the result was further confirmed by six in-house samples from two gastric cancer patients and five in-house samples from two colorectal cancer patients with different tumor purities. Furthermore, the metastasis mechanism of gastric cancer may be incorrectly characterized as numbers of mutation and tumor purities of 248 lymph node metastatic (N + M0) samples were both significantly lower than those of 121 non-metastatic (N0M0) samples (*p* < 0.05, Wilcoxon rank-sum test). Similar phenomena were also observed that tumor purities could confound the analysis of histological subtypes of cancer and the identification of microsatellite instability status (MSI) in both gastric and colon cancer. Finally, we suggested that the higher tumor purity, such as above 70%, rather than 60%, could be better to meet the requirement of mutation calling. In conclusion, the influence of tumor purity on the genomic mutation profile and pathological analyses should be fully considered in the further study.

## Introduction

Somatic mutation is accumulated during tumor development, which is commonly believed to play an important role in revealing the mechanism of carcinogenesis (Stratton et al., 2009; Stratton, 2011; Nik-Zainal et al., 2012). Recently, through sequencing analysis of cancer genomes, considerable advancements have been made in identifying cancer genes with “driver” mutation, such as TP53 (Moon et al., 2019), KRAS (Polom et al., 2019), BRAF (Yang et al., 2018a), EGFR (Paez et al., 2004), and PIK3CA (Harada et al., 2016). They provide insights into understand cancer development, find targets for therapeutic intervention (Alexandrov et al., 2013a, b) and develop diagnostic biomarkers. However, it has been reported that the identification of somatic mutation may be influenced by tumor purity (Koboldt et al., 2012; Cibulskis et al., 2013). As is known to all, tumor tissues collected patients contain not only tumor cells, but also non-tumor cells, e.g., infiltrating stromal cells, immune cells, fibroblasts and normal cells (Joyce and Pollard, 2009), which could dilute the purity of tumor cells. Specifically, DNA from tumor samples are inevitably contaminated with non-tumor DNA. Various tumor purities might affect mutation detections through disturbed the numbers of mutated read (Raphael et al., 2014), and consequently affect the biological interpretations of genomic analyses (Aran et al., 2015).

Several approaches have been proposed to reduce the influence of tumor purity on mutation detection. For example, most studies generally require samples with at least 60% of tumor nuclei. However, the threshold of tumor purity might remain to be further evaluated (Aran et al., 2015). Practically, it is often difficult to obtain some cancer samples with sufficient tumor purity, such as diffuse gastric cancer and pancreatic adenocarcinomas. The laser capture microdissection (LCM) is commonly used to isolated pure tumor cells from tumor tissues (Espina et al., 2006), but it is cost and time consuming, which makes it difficult to be widely used in clinical scenes. Meanwhile, other collection technologies have been reported to isolate pure or putative tumor cells from tumor tissues. For example, DEPArray technology could isolate putative tumor cells from cancer samples (Lee et al., 2018), but it is difficult to handle large number of cells from large volume of cancers because of sorting time and the expenses (Lee et al., 2018). Furthermore, several algorithms have been proposed to evaluate tumor purities based on the copy number ploidy variations (Carter et al., 2012), methylation (Zheng et al., 2014), or expression levels of signature genes (Yoshihara et al., 2013). However, these tumor purities commonly reflect the average proportion of various cell types or are biased to a certain cell type. And the measurements of genes are sensitive to experimental batch effects (Leek et al., 2010; Oesper et al., 2014). The evaluation and correction of tumor purity is very hard and the golden standard is still dependent on the pathologists. Therefore, it is necessary to fully evaluate the influence of tumor purity on the analysis of genome mutation profile.

Gastric cancer is one of the common malignant tumors (Siegel et al., 2017). Tumor progression of gastric cancer, e.g., metastasis or post-surgery relapse, is the main death cause, and the tumor-node-metastasis (TNM) staging is an important indicator for tumor progression, which T represents primary tumor, N represents metastasis of regional lymph nodes and M represents distant metastasis of cancer. Based on the TNM system, the absence or presence of lymph node metastasis is identified as N0M0 or N + M0. Meanwhile, according to the Lauren’s pathological classification, gastric cancer could be distinguished as intestinal, diffuse, or mixed subtypes (Shah et al., 2011). Compared with intestinal subtype, diffuse subtype has a different pattern of spread and behavior with a worse prognosis (Shah et al., 2011). The TNM staging system and the pathological classification are always used to determine the treatment strategies for gastric cancer patients. Besides, the microsatellite instability (MSI) status is another indicator for determining the treatment regimen in gastric cancer and colon cancer, which patients with high level of MSI (MSI-H) are less likely to benefit from the 5-Fu-based chemotherapy (Ilson, 2018). The MSI status were commonly identified by using immunohistochemistry and polymerase chain reaction (PCR), which measured the expressions of putative genes or the mutations of putative sites. However, molecular analyses between N0M0 and N + M0, or between diffuse and intestinal subtypes, or the identification of MSI status, may be affected by various tumor purities.

In this study, mainly using public gastric cancer samples from The Cancer Genome Atlas (TCGA) for example, the influence of tumor purity on mutation detection, pathological subtypes and the identification of MSI status were evaluated. Moreover, the biased influences were further evaluated in other nine cancers from TCGA and the in-house samples with different tumor purities from the same cancer patients. To obtain the robustly biological interpretations of genomic and pathological analyses, we suggested that the biased influences of various tumor purities should be fully considered.

## Materials and Methods

### Data and Pre-processing

#### Public Data and Pre-processing

The mutation profiles called by four algorithms (MuSE, MuTect2, SomaticSniper, and VarScan2) and the clinical information of stomach adenocarcinoma (STAD) samples were downloaded from TCGA (Table 1).^1^ Generally, multiple slides which were sampled from the top to bottom of the same tumor tissue were collected. Each slide was consisted of tumor cells and non-tumor cells. The percent of tumor nuclei in each slide was evaluated by pathologists. According to the report by Yoshihara et al. (2013), the tumor purity of a sample was the arithmetic mean percent of tumor nuclei in all slides. If the information of percent of tumor nuclei in one of the multiple slides was unavailable or the percent of tumor nuclei of all slides are zeros, the sample is excluded. Moreover, the mutation profiles and corresponding clinical information of other nine cancer types, included breast invasive carcinoma (BRAC), colorectal carcinoma (CRC), glioblastoma multiforme (GBM), brain lower grade glioma (LGG), liver hepatocellular carcinoma (LIHC), lung adenocarcinoma (LUAD), lung squamous cell carcinoma (LUSC), pancreatic adenocarcinoma (PAAD), and prostate adenocarcinoma (PRAD) were also downloaded, respectively. And 723 cancer genes were downloaded from the COSMIC database (Tate et al., 2019),^2^ which were used to analyze the influences of tumor purity on mutation callings of cancer genes.

**TABLE 1:** Description of the number of public data/samples used in this study.

Cancer type	Sample size			
	MuSE	MuTect2	SomaticSniper	VarScan2
STAD	432	436	426	432
BRCA	979	982	970	981
CRC	534	534	535	534
GBM	389	389	383	388
LGG	502	504	497	503
LIHC	361	363	360	363
LUAD	504	508	497	502
LUSC	485	487	482	485
PAAD	161	169	140	150
PRAD	472	486	456	475

### In-house Data and Measurement

Six surgical resection specimens from two gastric cancer patients were measured by whole-exome sequencing with mean depth of 80–100×. For each patient, three specimens were sampled in three different locations, whose diameters of tumor tissues were at least 50 mm, respectively. The tumor purities of six samples, measured by pathologists, ranged from 26.5 to 92.5%, as shown in Table 2. Meanwhile, five surgical resection specimens collected from two colorectal cancer patients in our previous study were used to validate the influence of tumor purity on mutation detection (Yan et al., 2019). The tumor purities of five colorectal cancer samples ranged from 40 to 100% (Table 2). This study was approved by the institutional review boards of all participating institutions, and written consent forms were obtained from all participants.

**TABLE 2:** The tumor purities of in-house gastric cancer and colorectal cancer samples.

Patient	Position A (%)	Position B (%)	Position C (%)
GC-1	92.50	72.50	26.50
GC-2	88.00	56.50	33.00
CRC-1	100.00	100.00	40.00
CRC-2	70.00	40.00	–

Afterward, according to the manufacture’s protocol, total DNA was isolated from the fresh frozen gastric tumor tissues and the generated raw whole-exome sequencing files (.fastq) were preprocessed using Trimmomatic (Bolger et al., 2014), and the reference genome (GRCh37) was used to align reads using Burrows-Wheeler aligner (BWA; Li and Durbin, 2009). Finally, the mutations were called using default parameters. Mutations included single nucleotide variation (SNV), indel (insertion and deletion, less than 50 bp) in this study. And they were filtered to exclude the mutation sites of germline risk based on gnomAD variant dataset file.^3^ Only those SNVs which were identified as mutations were further analyzed.

### Statistical Analysis

The spearman rank correlation analysis was used to assess the correlation between numbers of mutation and corresponding tumor purities in tumor samples. The wilcoxon rank-sum test was used to assess the difference of tumor purities (or numbers of mutation) between two groups of samples. And the fisher exact test was used to evaluate the significance of mutation frequencies of genes between high-purity and low-purity samples or between N0M0 and N + M0 samples. N0M0 and N + M0 represent non-metastatic samples and lymph node metastatic samples of gastric cancer, respectively. The hypergeometric test and cumulative binomial test were used to assess the impact of sample size on the correlation between numbers of mutation and tumor purities, respectively.

## Results

### Tumor Purity Confounds Mutation Detection

Taken gastric cancer as an example, we firstly analyzed the associations between numbers of mutation called by four mutation calling algorithms (MuSE, MuTect2, SomaticSniper, and VarScan2) and corresponding tumor purities, respectively. Tumor purities of gastric cancer samples distributed dispersedly, ranging from 5 to 100%. The tumor purity of about 72% gastric cancers were higher than 70%. The results showed that numbers of mutation called by MuSE and SomaticSniper algorithms were significant positively correlated with tumor purities (*p* = 2.22e-05 for MuSE and *p* = 1.84e-05 for SomaticSniper). Similar results were also observed in numbers of mutation called by MuTect2 (*p* = 1.00e-04) and VarScan2 (*p* = 7.73e-06) algorithms which are implanted the correction parameters of tumor purity. Notably, the significantly positive correlation between numbers of mutation and tumor purities in other nine cancer types could also be observed (Table 3). These results suggested that mutation detections might be significantly influenced by various tumor purities.

**TABLE 3:** The *p*-values of spearman’s rank correlations between tumor purities and numbers of mutation in other nine cancer types.

Cancer types	MuSE	MuTect2	SomaticSniper	VarScan2
BRCA	2.00e-04*	1.02e-02*	1.47e-06*	3.00e-04*
CRC	7.23e-02	2.03e-01	1.52e-04*	9.35e-02
GBM	3.42e-02*	1.16e-05*	1.66e-01	4.67e-02*
LGG	7.25e-08*	5.49e-06*	6.75e-10*	1.45e-08*
LIHC	5.65e-02	1.22e-01	5.20e-03*	2.98e-02*
LUAD	4.17e-02	8.84e-01	1.54e-02*	3.03e-01
LUSC	9.35e-05*	5.30e-03*	3.07e-08*	8.69e-05*
PAAD	2.34e-02*	3.42e-02*	7.40e-03*	6.71e-02
PRAD	4.00e-04*	4.00e-04*	3.61e-07*	7.05e-05*

*Represented the significance of *p*-value.

**FIGURE 1:**
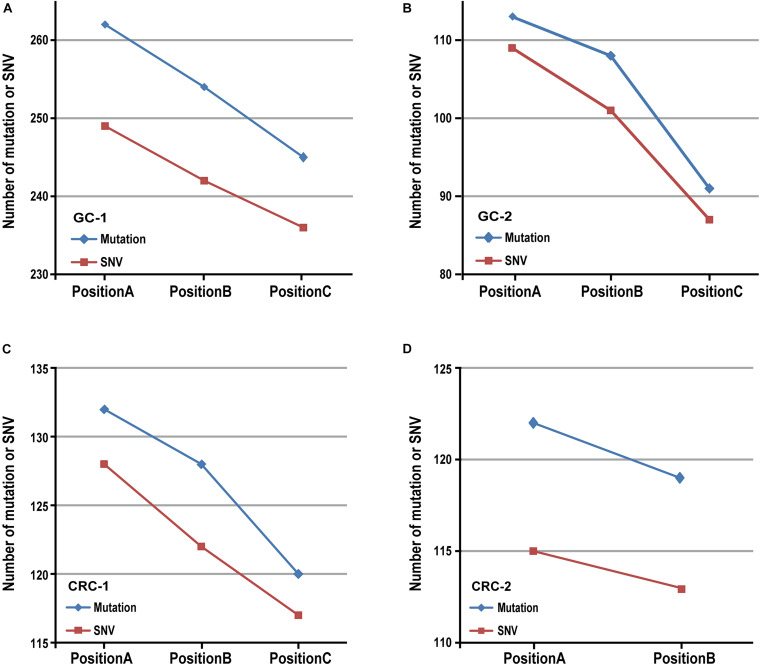
The result of mutation calling in different sampling positions. The number of mutation (or SNV) decreased as tumor purities decreased in two patients with gastric cancer **(A,B)** and two patients with colorectal cancer **(C,D)**.

Then we verified the influence of tumor purity on mutation detection using MuTect2 algorithm in six in-house gastric tumor samples, which were sampled from three different locations with different tumor purities from each gastric cancer patient. The results showed that, for the samples from the same patient, the numbers of mutation decreased as the tumor purities decreased, as shown in Figures 1A,B. Similar results were also observed in five in-house colorectal tumor samples collected from two patients, as shown in Figures 1C,D. The results further confirmed that various tumor purities might affect numbers of mutation. Moreover, similar results were observed in numbers of mutation detected by the Varscan2, SomaticSniper and MuSE algorithms, respectively, which decreased with the tumor purities, as shown in Supplementary Table 1.

Additionally, we further analyzed the numbers of mutated reads aligned to each mutation site in measured gastric cancer samples. For GC-1 patient, among 19 SNVs that were identified in samples with tumor purities of 92.50 and 72.50%, 15 SNVs were not detected in sample with the lowest tumor purity of 26.50%. Nevertheless, they were aligned to several mutated fragments (14 SNVs: 1--4 reads and 1 SNV: 6 reads). Similarly, 14 SNVs were not identified as mutations in the position C with 33% of tumor purity for GC-2 patient, but they were also aligned to several mutated fragments (1--5 reads). Those unidentified mutation sites in the position C of two patients included the genes *FBXO11* and *XPO1*, which were identified as cancer genes in the COSMIC database,^4^ shown in Table 4. These results indicated that the artificially low mutation burden might result from low tumor purities.

**TABLE 4:** Mutations of *FBXO11* and *XP01* in different sampling positions.

Patient/gene	Sites	Mutation type	Mutation site	Mutation reads	Aligned reads
GC-1/FBXO11	PositionA	disruptive_inframe_del	c.11_37del	5	57
	PositionB	disruptive_inframe_ del	c.11_37del	8	51
	PositionC	no	no	1	81
GC-2/XP01	PositionA	missense	c.1426T > C	7	68
	PositionB	missense	c.1426T > C	11	100
	PositionC	no	no	0	98

### Tumor Purity Confounds the Mutation Differences Between Metastasis and Non-metastasis of Gastric Cancer

**FIGURE 2:**
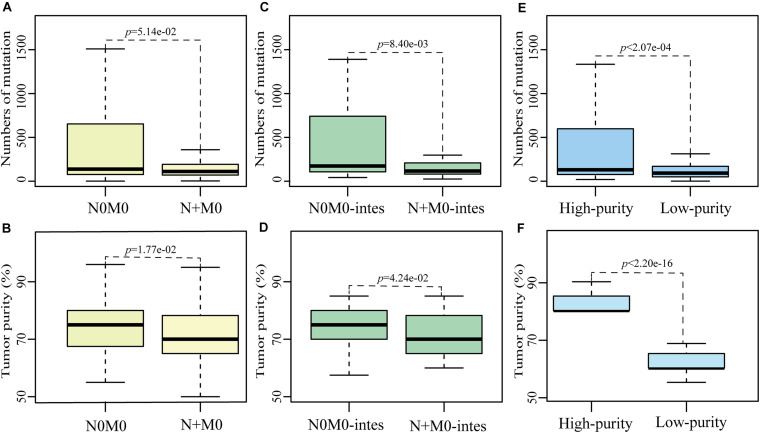
The influence of tumor purity on mutation detection between N0M0 and N + M0 samples. The differences of number of mutation or tumor purity between N0M0 and N + M0 samples **(A,B)**, between N0M0 (N0M0-intes) and N + M0 (N + M0-intes) samples in intestinal gastric cancer **(C,D)**, and between high-purity and low-purity samples **(E,F)**. Outline points were deleted.

Based on the non-synonymous mutation data of primary gastric cancer samples from TCGA database, which were called by MuTect2 algorithm, we found that the numbers of mutation in 248 N + M0 samples tended to be significantly less than those in 121 N0M0 samples (*p* = 5.14e-02, Wilcoxon rank-sum test, Figure 2A). Then we compared the differences of multiple clinical factors between two subgroups, including age, gender, tumor purity and grade, and found that only tumor purity was significantly different between two subgroups. The tumor purities in N + M0 samples were significantly lower than those in N0M0 samples (*p* = 1.77e-02, Wilcoxon rank-sum test, Figure 2B). In order to remove the biased influence of sample sizes, we randomly selected 121 samples from 248 N + M0 samples and compared tumor purities and numbers of mutation between 121 N0M0 and 121 N + M0 samples. The random experiment was repeated 1,000 times. The result showed that there were 546 times of significantly different tumor purities between N0M0 and N + M0 samples, 388 times of significantly different numbers of mutation, and 246 times that tumor purity and number of mutation were both significantly different (all *p* < 0.05, Wilcoxon rank-sum test). The results were not happened randomly (*p* < 1.00e-16, hypergeometric test), which indicated that the biased sample sizes could not be the main cause of mutation differences between N0M0 and N + M0 samples. Removing diffuse gastric tumor samples with high heterogeneity, similar phenomena were also observed in intestinal gastric cancer that numbers of mutation in 115 N + M0 samples were significantly less than those in 46 N0M0 samples (*p* < 8.40e-03, Wilcoxon rank-sum test, Figure 2C), and tumor purities in 115 N + M0 samples were also significantly less than those in 46 N0M0 samples (*p* < 4.24e-02, Wilcoxon rank-sum test, Figure 2D). The results indicated that the difference of numbers of mutation between N0M0 and N + M0 may be mainly caused by the variations of tumor purity. The lower tumor purities of N + M0 samples could lead to the artificially lower mutation burden than that of N0M0 samples.

Meanwhile, we also found the mutation frequency of 1,184 genes were significantly different between N0M0 and N + M0 samples (*p* < 0.05, Fisher’s exact one-side test). Subsequently, we divided the primary gastric tumor tissues into two groups according to tumor purities. Totally, 129 samples whose tumor purities were at least 80% were divided into the high-purity group, while 127 samples whose tumor purities were less than 70% were divided into the low-purity group. The information of low- and high-purity samples in different categories was shown in Table 5. The numbers of mutation in low-purity samples were significantly lower than those in high-purity samples (*p* < 0.05, Wilcoxon rank-sum test, Figures 2E,F). Similarly, the mutation frequencies of 1,247 genes were significantly different between high-purity and low-purity groups (*p* < 0.05, Fisher’s exact one-side test). There were 184 genes overlapped with the 1,184 genes of differentially mutated frequency between N0M0 and N + M0 samples, of which 182 genes had significantly higher mutation frequency in both N0M0 samples and high-purity samples. Gene *SLC3A2* and *APC*, which were associated with metastasis and neoplasia (Ghatak et al., 2017; Wang et al., 2017), were included. These results indicated that various tumor purities had an impact on mutation differences between N0M0 and N + M0 samples, which might confound the interpretation of metastasis mechanism for gastric cancer.

**TABLE 5:** The number of low- and high-purity samples in different categories.

Sample size	High_purity ≥ 80%	Low_purity < 70%
All(436)	129	127
N0M0(121)	46	31
N + M0(248)	61	80
N + M0-intes(115)	28	40

### Tumor Purity Confounds the Molecular Analysis of Gastric Cancer Subtypes

**FIGURE 3:**
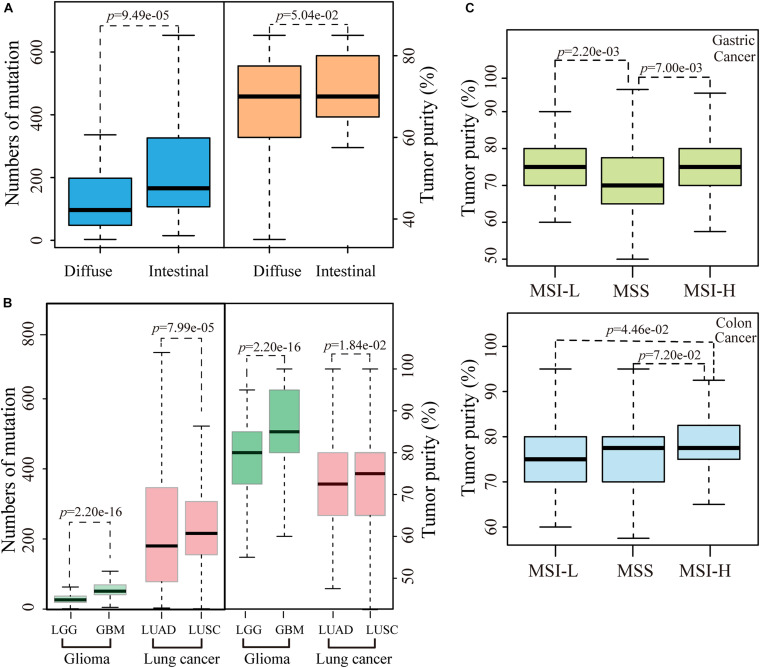
The influence of tumor purity on MSI status and pathological subtypes. **(A)** The differences of number of mutation or tumor purity between the diffuse and intestinal gastric cancer. **(B)** The differences of number of mutation or tumor purity between GBM and LGG or between LUAD and LUSC. **(C)** The differences of tumor purity between different MSI status both in gastric and colon cancer. Outline points were deleted.

We then evaluated the influence of tumor purity on the mutation analysis between the diffuse and intestinal histological subtypes of gastric cancer. No significant difference of tumor purity was observed between 70 diffuse samples and 190 intestinal samples (*p* = 1.45e-01, Wilcoxon rank-sum test). However, after excluding five intestinal and four diffuse unrepresentative samples that only had one slide with more than 90% of tumor purity, the tumor purities of 66 diffuse samples tend to be significantly lower than those of 185 intestinal samples (*p* = 5.04e-02, Wilcoxon rank-sum test), while numbers of mutation in diffuse subtype were significantly less than those in intestinal subtype (*p* = 9.49e-05, Wilcoxon-rank test), as showed in Figure 3A. Furthermore, similar phenomena that the significant differences of tumor purities and numbers of mutation between the histological subtypes of lung cancer (including LUAD and LUSA) or glioma (including GBM and LGG) were also observed, respectively, as shown in Figure 3B. The results suggested the various tumor purities might confound the mutation differences between different histological subtypes of cancer.

### Tumor Purity Confounds the Identification of MSI Status

We further evaluated the influence of various tumor purities on the identification of a known pathological biomarker, the MSI status, which is commonly used to determine the follow-up treatment regimen for gastric and colon cancer patients. According to the MSI status of gastric cancer, the tumor purities of 241 samples with stable level of MSI were significantly lower than both 72 MSI-H samples and 56 low level of MSI (MSI-L) samples, respectively (all *p* < 0.05, Wilcoxon rank-sum test, Figure 3C). Compared with the distribution of tumor purities of gastric cancer samples, the tumor purities of colon cancer samples distributed narrowly, and 86% of the colon cancer samples were with ≥70% of tumor purities. No significant correlation was observed between number of mutation and tumor purity in colon cancer. However, the tumor purities of 83 MSI-H samples were significantly higher than those of 82 MSI-L samples (*p* = 4.46e-02, Wilcoxon rank-sum test) and tentative significantly higher than those of 291 samples with stable level of MSI (*p* = 7.20e-02, Wilcoxon rank-sum test), respectively, as shown in Figure 3C. The above results suggested that various tumor purities might confound the identification of MSI status.

### An Appropriate Threshold of Tumor Purity for Mutation Calling

Finally, we took gastric cancer as an example to identify an appropriate tumor purity for mutation calling. According to the at least 60% of tumor purity required in most researches, we firstly removed the gastric cancer samples with tumor purity less than 60%, and observed that numbers of mutation called by four algorithms were still significant positively correlated with tumor purities (*p* < 0.05, Table 6). These results indicated that higher tumor purity may be needed for mutation calling. Then we analyzed samples with higher than 70% of tumor purity. No significant correlation was observed between tumor purity and number of mutation, except for SomaticSniper algorithm. Moreover, similar results that non-significant correlation between tumor purities and numbers of mutation were observed in other nine cancer types, except for LGG (Table 6).

**TABLE 6:** The *p*-values of spearman’s rank correlation between tumor purity higher than 60 or 70% and number of mutation.

Cancer types	MuSE	MuTect2	SomaticSniper	VarScan2
**Tumor purity ≥ 60%**				
STAD	1.24e-04*	1.40e-03*	7.05e-05*	8.44e-05*
BRCA	1.40e-03*	4.68e-02*	5.57e-05*	4.80e-03*
CRC	1.08e-01	2.64e-01	5.48e-04*	1.14e-01
GBM	8.00e-02	5.37e-05*	1.96e-01	1.07e-01
LGG	1.26e-06*	1.88e-05*	2.59e-08*	1.72e-07*
LIHC	5.65e-02	1.22e-01	5.20e-03*	2.98e-02*
LUAD	7.03e-01	8.10e-01	4.01e-02*	5.49e-01
LUSC	6.85e-04*	1.58e-02*	5.63e-07*	4.54e-04*
PAAD	NAN	NAN	NAN	NAN
PRAD	7.61e-06*	3.31e-05*	4.10e-07*	3.29e-05*
**Tumor purity > 70%**				
STAD	1.43e-01*	4.19e-01*	3.17e-02*	1.29e-01*
BRCA	1.78e-01	5.47e-01	1.76e-02*	7.00e-02
CRC	3.02e-01	5.69e-01	6.30e-03*	3.75e-01
GBM	1.14e-01	9.54e-05^#^	3.60e-01	1.34e-01
LGG	7.00e-03*	1.87e-02*	7.40e-03*	1.40e-03*
LIHC	1.76e-01	3.71e-01	3.58e-02*	1.42e-01
LUAD	3.74e-01	6.55e-01	4.80e-02*	4.17e-01
LUSC	3.40e-01	3.05e-01	1.84e-01	4.43e-01
PAAD	NAN	NAN	NAN	NAN
PRAD	8.21e-02	2.25e-01	2.61e-02*	7.45e-02

^#^ and * represented non-significant and significant *p*-value (< 0.05) calculated by spearman rank correlation, respectively. NAN represented that the *p*-value was not calculated due to small sample size.

In order to remove the influence of sample size, the same size of gastric samples with above 70% of tumor purity were randomly selected from samples with ≥60% of tumor purity and the correlations between tumor purities and numbers of mutation were calculated. The random experiment was repeated 1,000 times. Finally, a cumulative binomial test was used to assess the significance of positive correlation in the 1,000 random experiments. The results showed that 65.50% of 1,000 random experiments were significant correlations in Mutect2 algorithms and more than 80% of 1,000 random experiments were significant correlations in other three algorithms, respectively (all *p* < 0.05, binomial test, Supplementary Table 2). Similar results of random experiments were also observed in other multiple cancer types (Supplementary Table 1). These results indicated that the sample sizes could not be the major factor of correlation between number of mutation and tumor purity. In a word, above 70% of tumor purity, rather than 60%, might be better to meet the requirement of mutation calling.

## Discussion

As showed in this study, numbers of mutation and tumor purities were significantly positive correlation in gastric cancer and other nine cancer types, regardless of calling algorithms. The lower tumor purities may lead to the artificially lower mutation burden, which may consequently cause the misleading biological interpretation of metastasis mechanism, pathological subtypes, as well as pathological biomarker analyses. Finally, we suggested that above 70% of tumor purity could be better to meet the requirement of mutation callings.

Moreover, gene *FBXO11*, *XPO1*, *SLC3A2*, and *APC*, whose mutation detections may be affected by various tumor purities in gastric cancer, were closely related with cancer occurrence and development. For examples, protein FBXO11 has both the E3 ubiquitin ligase and methyltrasferase activity, which could facilitate epithelial-mesenchymal transition (EMT), promote PI3K/AKT pathway activation, and regulate metastasis and apoptosis in human cancer (Kim et al., 2018, 2020; Sun et al., 2018). Protein XPO1 is positively correlated with cell proliferation and growth transformation, and negatively correlated with poor survival outcomes, which could be a promising molecular target in gastric cancer (Subhash et al., 2018; Gruffaz et al., 2019; Sexton et al., 2019). Protein SLC3A2 is associated with the migration and invasion of tumor cells (Wang et al., 2017), which is a potential biomarker for molecular imaging-based detection of gastric cancer (Yang et al., 2012). Gene *APC*, which is involved in Wnt/β-catenin signaling pathway, has been reported to be associated with tumorigenesis, tumor metastasis and resistance (Yang et al., 2018b).

Currently, many studies have been proposed that tumor mutation burden (TMB) could predict the response to immunotherapy (Goodman et al., 2017; Qin et al., 2018), which patients with high TMB commonly responds better to immunotherapy than patients with low TMB. However, due to the differences in surgical sampling or biopsy sites of tumor tissue, the TMB or the pathologic biomarkers, such as PDL-1 (Anagnostou et al., 2017; Qin et al., 2018), could be affected by various tumor purities. For this problem, some researches proposed to increase the sequencing depth to reduce the false negatives from low tumor purity, but it might also sharply increase the false positives of mutation detection, work burden and cost.

Additionally, for the threshold of tumor purity, TCGA originally required at least 80% of tumor nuclei (Aran et al., 2015), but it is generally difficult to collect enough amount of samples. Then, this threshold was later reduced to 60% as the RNA-seq technology developed. And most current studies set the threshold as 60%. However, the research by Dvir Aran et.al (Aran et al., 2015) indicated that the impact of 60% of tumor purity on the interpretation of genomic analyses remained to be evaluated. Our results in ten cancer types showed that, above 70% of tumor purity, rather than 60%, might be better to meet the requirement of mutation calling and obtain relatively sufficient and reliable mutation profiles. Certainly, a novel mutation detection algorithm for tumor sample with low purity should be developed as soon as possible.

A major limitation is that the tumor heterogeneity, pathological subtypes, and the colonal selection of mutations do affect mutation callings during the process of tumor occurrence and development (Gerlinger et al., 2012), which could not be excluded in this study. However, our study revealed that there were universal significantly correlations between numbers of mutation and tumor purities in ten cancer types. Although the sample size of in-house data is small in this study, the low tumor purities resulted in less mutations that were further demonstrated in six gastric cancer samples from two patients and five colorectal cancer samples from two patients with different tumor purities. That suggested that numbers of mutation were influenced by tumor purities regardless of tumor types and the influence of tumor purity on number of mutation should be noticed.

In conclusion, the influences of various tumor purities on mutation detection and pathological analyses should be fully considered in further analysis. And we suggested that more than 70% of tumor purity could be better to meet the requirement of mutation calling.

## Data Availability Statement

The in-house data used and analyzed during the current study is available from the corresponding authors upon reasonable request.

## Ethics Statement

The studies involving human participants were reviewed and approved by The Affiliated Union Hospital of Fujian Medical University. The patients/participants provided their written informed consent to participate in this study. Written informed consent was obtained from the individual(s) for the publication of any potentially identifiable images or data included in this article.

## Author Contributions

ZG conceived the idea. LA and JC conceived and designed the experiments and wrote the manuscript. JH designed the experiments and made figures. SSW and ZXZ searched the data and participated in the statistical analysis. QZG and HDY helped in interpreting the results and writing the manuscript. JL helped in writing the manuscript. All authors approved the final version.

## Conflict of Interest

The authors declare that the research was conducted in the absence of any commercial or financial relationships that could be construed as a potential conflict of interest.

## Footnotes

1 http://cancergenome.nih.gov/

2 https://cancer.sanger.ac.uk/cosmic/

3 https://gnomad.broadinstitute.org/downloads

4 https://cancer.sanger.ac.uk/cosmic/

## Supplementary Material

Supplementary Table 1 Numbers of mutation called by other three algorithms.

Supplementary Table 2 The number of significant *p*-value in 1000 random experiments.
