# Supplementary material for: Biased Influences of Low Tumor Purity on Mutation Detection in Cancer
Source: Front Mol Biosci. 2020 Dec 23;7:533196. doi: 10.3389/fmolb.2020.533196 (PMC7785586; doi:10.3389/fmolb.2020.533196)
Supplement: Supplementary Table 1 — Numbers of mutation called by other three algorithms. [file Table_1.DOC]

| Supplementary Table S1. Numbers of mutation called by other three algorithms | | | |
| --- | --- | --- | --- |
| Sample/Purity | Varscan2 | SomaticSniper | MuSE |
| GC1_A(92.5%) | 164 | 180 | 316 |
| GC1_B(72.5%) | 163 | 170 | 297 |
| GC1_C(26.5%) | 142 | 157 | 217 |
| GC2_A(88.0%) | 750 | 761 | 129 |
| GC2_B(56.5%) | 736 | 721 | 34 |
| GC2_C(33.0%) | 556 | 457 | 19 |
| CRC1_A(100%) | 484 | 537 | 163 |
| CRC1_B(100%) | 478 | 557 | 150 |
| CRC1_C(40%) | 418 | 494 | 160 |
| CRC2_A(70%) | 331 | 330 | 255 |
| CRC2_B(40%) | 299 | 315 | 237 |
