# Supplementary material for: Biased Influences of Low Tumor Purity on Mutation Detection in Cancer
Source: Front Mol Biosci. 2020 Dec 23;7:533196. doi: 10.3389/fmolb.2020.533196 (PMC7785586; doi:10.3389/fmolb.2020.533196)
Supplement: Supplementary Table 2 — The number of significant p-value in 1000 random experiments. [file Table_2.DOC]

| **Supplementary Table S2. The number of significant *p*-value in 1000 random experiments** | | | | |
| --- | --- | --- | --- | --- |
| Algorithm | MuSE | MuTect2 | SomaticSniper | VarScan2 |
| STAD | 811* | 655* | 843* | 803* |
| BRCA | 764* | 426^#^ | 953* | 678* |
| LUSC | 717* | 463^#^ | 961* | 752* |
| PRAD | 910* | 864* | 968* | 923* |

Note: ^*^ represented the significance of *p*-value (< 0.05) using cumulative binomial distribution model; ^#^ represented *p*-value is not significant.
